# Prenatally Diagnosed Isolated Coronary Arterial Fistula Leading to Severe Complications at Birth

**DOI:** 10.1155/2018/2509502

**Published:** 2018-03-26

**Authors:** A. Wacker-Gussmann, T. Esser, S. M. Lobmaier, M. O. Vogt, E. Ostermayer, R. Oberhoffer

**Affiliations:** ^1^Institute of Preventive Pediatrics, Faculty of Sport and Health Sciences, and German Heart Center, Department of Pediatric Cardiology and Congenital Heart Defects, Munich, Germany; ^2^Outpatient Clinic of Prenatal Diagnostics, Munich, Germany; ^3^Department of Obstetrics and Gynecology, Klinikum rechts der Isar, Technical University of Munich, Munich, Germany; ^4^German Heart Center, Department of Pediatric Cardiology and Congenital Heart Defects, Munich, Germany

## Abstract

Prenatal diagnosis of a huge coronary artery fistula between the left coronary artery and the right ventricle was made by Doppler echocardiography at 22 weeks of gestation. Progression of the dilated fistula was monitored throughout pregnancy. The size of the fistula increased enormously up to 11 mm. Death occurred at birth. Monitoring of these fetuses is essential as severe complications can occur during pregnancy or at birth.

## 1. Introduction

Fetal echocardiography offers possibilities to identify structural heart defects prenatally. However, visualization of coronary arteries and their blood flow remains difficult because of the small size of the coronary vessels and the movement of the fetus. Previous published data specified an incidence of isolated coronary arterial fistulas (CAFs) in 1 : 50,000 births and a prevalence of ventricular aneurysm in 0.5 : 100,000 births [1]. Mainly single rather than multiple coronary arteries are involved. We report about a prenatal diagnosis of a huge CAF between the left coronary artery and the right ventricle, which was made by Doppler echocardiography at 22 weeks of gestation.

## 2. Case Presentation

**Figure 1:**
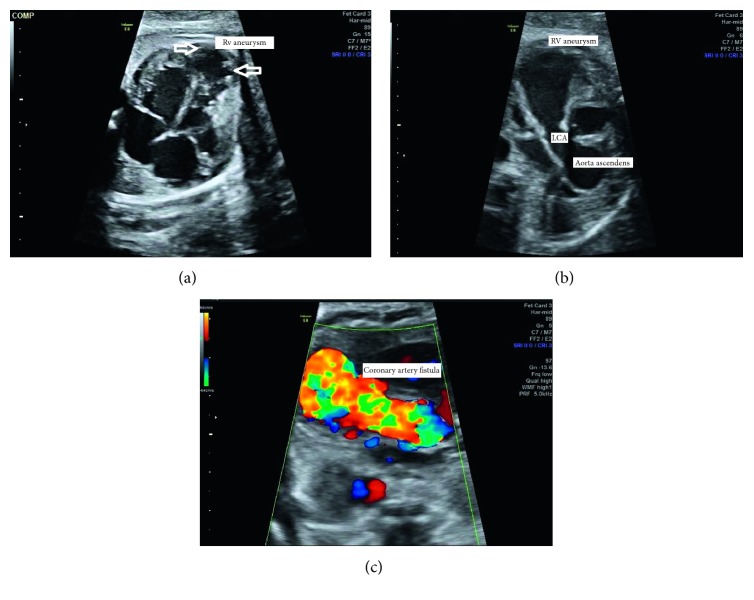
(a) A 4-chamber view: bizarre configuration of the right ventricle (RV), large aneurysm at the apex of the RV, and the remaining functional part, bulked myocardium. (b) Modified short axis with ascending aorta in the middle, giving rise to the giant coronary artery (LCA), which drains into the right ventricular aneurysm. (c) Entire course of the coronary artery fistula and bidirectional flow in the left coronary artery.

A 29-year-old pregnant woman was referred at 22 weeks of gestation because the fetal heart was suspected to be abnormal in a routine ultrasound examination. The fetus was appropriate for age, and no other malformation was found. A high-resolution ultrasound device (Voluson E8 Expert, GE Healthcare, Munich) was used for ultrasound examination of the fetal heart. The fetal heart rate was within normal range. The right ventricle was dilated due to an apical aneurysm, and the contractility of the right ventricle was reduced. The function of the left ventricle seemed to be slightly reduced. Objective measurement of fractional shortening was not possible due to the disturbed right ventricular morphology. A left CAF (diameter 2.9 mm) draining into the aneurysmatic part of the right ventricle was visualized by color Doppler ultrasound. Pulsed Doppler recordings revealed characteristic bidirectional flow in the fistula. Turbulent diastolic reversed flow was detected in the aortic arch resulting from the blood steal phenomenon via CAF into the right ventricular aneurysm (Figure 1). Genetic testing was normal. The poor prognosis and likelihood of intrauterine or perinatal death were discussed with the parents; however, they decided to continue pregnancy. During pregnancy, there was an increase in the size of the fistula, and it became dilated up to 11.5 mm at 37 weeks of gestation (Table 1). The size of the aneurysmatic right ventricle area became larger, and cardiomegaly developed at 35 weeks of gestation. The remaining functional right ventricle became smaller and was difficult to differentiate from the aneurysmatic part of the right ventricle, resulting in a bizzare formation of the right ventricular entity. No tricuspid regurgitation, pericardial effusion, or hydrops developed. The proportion of the aorta and pulmonary artery was normal and remained balanced. Brain-sparing physiology on middle cerebral arterial Doppler was not found. But there was a steal phenomenon, within the aortic arch and head-neck vessels due to the massive filling of the coronary arterial fistula draining blood away from the aortic root. Birth was planned in a center closely related to a major pediatric cardiac center. At 37 3/7 weeks of gestation, induction of labor was performed due to the obvious progression of the disease. Due to less fetal well-being, the child was born by cesarean section at 37 3/7 weeks of gestation in a tertiary obstetric center with standby of pediatricians specialized in pediatric cardiology. The birth weight was 3350 g, length 51 cm, Apgar score 1/3/2, and umbilical artery birth pH 7.29. Despite extremely prolonged resuscitation, the newborn died at 3 hours of life. Postmortem pathology was refused by the parents.

## 3. Discussion

CAFs are abnormal connections between a coronary artery and the heart or other blood vessels. Appearance of CAF is uncommon, if anatomy of the fetal heart is otherwise normal.

Most of the prenatally diagnosed CAFs have been reported to be asymptomatic. In some cases, small fistulas close spontaneously, whereas large fistulas, as in our presenting case, may increase in size and lead to severe complications [2]. Drainage into a low-pressure system can result in left-to-right shunts leading to heart failure, enlargement of the right chamber, myocardial ischemia from steal effects, and arrhythmia or embolisms [3]. Associated findings such as ventricular aneurysm, as presented in our case, or hydrops can occur. Treatment options are surgical and more recently transcatheter closures of the presenting fistula [4]. Transcatheter closures can result in similar efficacy and safety as surgical treatment. In our case, the right ventricular function seemed to be already decreased at the time of first presentation and became more reduced in its functional parts due to enlargement of the right ventricular fistula. At birth, the newborn was completely unstable and not advisable for any kind of therapeutic procedure. We speculate that death after birth in the presenting case was related to ischemia or low cardiac output due to the steal effects which have been balanced in utero due to different fetal hemodynamic entities. After birth, the usual cardiac output and peripheral resistance increase and decrease of the resistance of the lungs might have led to the augmented blood flow through the fistula into the right ventricle. This might have contributed to a reduced systemic and subendocardial perfusion, leading to subendocardial ischemia and finally low systemic perfusion.

In conclusion, CAF is a rare prenatal diagnosis without associated cardiac malformations but can dilate enormously and lead to ventricular aneurysms and heart failure in utero and immediately after birth. There is no broad experience in its treatment before birth at all.

## Figures and Tables

**Table 1:** Prenatal echocardiography: measurements of the fistula and right ventricular aneurysm.

	20 weeks of gestation	28 weeks of gestation	35 weeks of gestation	37 weeks of gestation
Fistula (mm)	2.9	4.4	8.2	11.5
Right ventricular aneurysm (mm)	9.5 × 7	16 × 11	15 × 27	17 × 34
